# Associations between cardiorespiratory fitness and diverticulitis in older adults

**DOI:** 10.1371/journal.pone.0275433

**Published:** 2022-09-29

**Authors:** Bong Kil Song, Joey M. Saavedra, Elizabeth C. Lefferts, Angelique G. Brellenthin, Duck-chul Lee

**Affiliations:** Department of Kinesiology, College of Human Sciences, Iowa State University, Ames, Iowa, United States of America; Bangor University, UNITED KINGDOM

## Abstract

**Objectives:**

Examine the independent and joint associations of cardiorespiratory fitness (CRF) and body mass index (BMI) with the prevalence of diverticulitis in older adults.

**Methods:**

476 older adults (61% Female; 71 ± 5 years) with no history of myocardial infarction, stroke, cancer, inflammatory bowel disease, or diabetes were included in this cross-sectional study. Diverticulitis cases were identified by self-reported physician diagnosis from the medical history questionnaire. Logistic regression was used to calculate the odds ratios (ORs) and 95% confidence intervals (CIs) of the prevalence of diverticulitis by tertiles of CRF and BMI category. CRF and BMI were further dichotomized into either “unfit” (the lowest one-third of CRF), “fit” (the upper two-thirds of CRF), “overweight/obese” (BMI ≥25.0 kg/m^2^), or “normal-weight” (BMI <25.0 kg/m^2^) to investigate the joint association of CRF and BMI with diverticulitis.

**Results:**

Thirty-five (7.4%) participants were identified as having diverticulitis. Compared with the lowest CRF tertile, the ORs (95% CIs) of diverticulitis were 0.52 (0.22–1.22) and 0.33 (0.12–0.94) in the middle and upper CRF tertiles, respectively, after adjusting for potential confounders. After further adjustment for BMI, the association was no longer significant with ORs (95% CIs) of 0.55 (0.23–1.33) and 0.37 (0.12–1.10) in middle and upper CRF tertiles, respectively. Compared with the normal-weight group, the ORs (95% CIs) of diverticulitis were 2.86 (1.05–7.79) and 2.98 (0.95–9.35) in the overweight and obese groups, respectively, after adjusting for possible confounders and CRF. Compared with the “unfit and overweight/obese” group in the joint analysis, the OR (95% CI) of diverticulitis was 0.16 (0.04–0.61) in the “fit and normal-weight” group.

**Conclusions:**

Older adults who maintain higher CRF and lower BMI may have significantly lower odds of diverticulitis, with the lowest odds found in the normal-weight and fit older adults.

## Introduction

Diverticulitis is a common gastrointestinal condition, impacting over 30% of older adults in North America [1], and increases the risk of cardiovascular disease [2]. The risk factors for diverticulitis include smoking; a diet high in saturated fat, low in fiber, or high in red meat; a high percent body fat; physical inactivity; and older age [1–7]. In fact, diverticulitis is the most common gastrointestinal disorder for which the diagnosis increases with age [6]. Although its causes are not fully understood, age-related factors such as slower metabolism, physical inactivity, hormonal changes, and reductions in cardiorespiratory fitness (CRF) may contribute to diverticulitis.

However, the potential associations of CRF with diverticulitis have yet to be elucidated, especially in an older adult population who has a higher prevalence of diverticulitis. One previous study found that vigorous physical activity, including running, which tends to improve CRF is inversely associated with reduced risk of diverticulitis. Moreover, the study reported that diverticular disease risk was also inversely related to cardiorespiratory fitness as measured by performance in a 10-km race [8].

Conversely, higher body mass index (BMI) is a well-known risk factor for diverticulitis and is well-supported by a broad base of evidence [7, 9, 10]. A meta-analysis, comprised of data from 1,636,777 adults, has shown an increase in the risk of diverticulitis with increasing BMI [9]. Although BMI is a well-established risk factor of diverticulitis, no study has examined the associations of BMI, independent of and combined with CRF, on the prevalence of diverticulitis in older adults. Examining the independent and combined associations of CRF and BMI with the prevalence of diverticulitis could provide novel information and reveal the relative importance of CRF or BMI and the potential additive benefits of both. Therefore, further research is needed to clarify the relationship between CRF and BMI on diverticulitis in the older adult population. This information could help inform future guidelines for older adults to lower the prevalence of diverticulitis.

The aim of this study is to investigate the independent and combined associations of CRF and BMI with diverticulitis in older adults.

## Materials and methods

### Study population

The Physical Activity and Aging Study (PAAS) is a prospective cohort consisting of older adults aged 65 years and above who participate in a series of laboratory visits involving the completion of standardized medical history questionnaires and comprehensive health and fitness assessments. Since the start of the study, 716 men and women have participated in PAAS. To minimize any potential confounding effects, we excluded 42 individuals reporting cardiovascular disease (myocardial infarction, stroke, or congestive heart failure), 112 individuals being treated for cancer, 13 individuals with a history of inflammatory bowel disease, and 53 individuals with a history of diabetes. A further 20 individuals with missing data for any covariates were excluded from this analysis. Therefore, 476 older adults (mean ± SD, age = 71.2 ± 5.4 years) were included in the present analyses. All participants provided written informed consent. This study was conducted in accordance with the Declaration of Helsinki, and the study was initially approved by the Iowa State University institutional review board in August 25, 2015 (IRB ID: 15–430).

### Data collection procedures

The baseline assessment consisted of two visits that were separated by a period of one week. At the first visit, participants provided information on their medical history and lifestyle factors (dietary information, smoking status, and alcohol consumption) as well as completed a cardiorespiratory fitness assessment. A week later, participants completed fasted assessments (no food, alcohol, or caffeine 12 hours before assessments), including height, weight, blood pressure, and a blood draw. Between the two visits, participants were given pedometers (Omron, Model HJ-321, IL, USA) to wear during waking hours for seven days to objectively measure PA levels (i.e., daily step counts). Participants wore the pedometers attached to their hips or pockets and removed only during underwater activities (e.g., swimming, showering) or sleeping. The pedometer has 7 days of memory and is reset at midnight every day and participants self-reported wear time. The pedometer data were considered valid if participants wore the device for more than 4 days with at least 10 hours per day following earlier studies [11–13]. The total number of steps per day was calculated by averaging the steps count on all valid days.

### Body mass index

Weight was measured in kilograms (kg), and height was measured in centimeters (cm) using a dual-function digital scale (SECA, Model 769, Chino, CA, USA). BMI was defined as the weight (kg) divided by height (meters squared). Participants were classified as normal weight, overweight, and obese using the World Health Organization (WHO) cut-points of <25.0 kg/m^2^, 25.0–29.9 kg/m^2^, and ≥30.0 kg/m^2^, respectively [14].

### Cardiorespiratory fitness (CRF)

Cardiorespiratory fitness (CRF) was assessed using a validated 400-meter walk test [15], and with the time taken (minutes) to complete the test as our indicator of CRF. Briefly, participants walked ten laps as fast as possible, without running, 20-meters apart. Participants were allowed to rest up to 60s if they remained standing. The maximum time allowed was 15 minutes, and all participants included in this study finished the test within that time frame. In older adults, the 400-m walking test is considered an appropriate and valid method to estimate CRF. Completion times from the 400-m walking test are associated with VO_2max_ from maximal (r = −0.79) and submaximal treadmill test (r = −0.66) in older adults [16, 17]. The 400-m walk test has previously demonstrated excellent reproducibility when tests were repeated a week apart (intraclass correlation coefficient [ICC], 0.95; 95% CI, 0.92–0.97) [16].

### Diagnosis of diverticulitis

Cases of diverticulitis were identified by self-reported physician diagnosis from the medical history questionnaire where participants were asked, "Have you ever been diagnosed with diverticulitis by a physician?" If yes, the year of diagnosis was obtained, which was also considered in diverticulitis diagnosis.

### Covariates

Information about medical history (hypercholesterolemia and hypertension), current medications (non-steroidal anti-inflammatory drugs [NSAIDs]), lifestyle factors (smoking status and alcohol consumption), and food intake (meals/week, vegetable cups/day, and fruit cups/day) were collected using the medical history questionnaire. Hypercholesterolemia was defined according to the self-reported physician diagnosis of hypercholesterolemia, the use of cholesterol medication (including statin), or low-density lipoprotein cholesterol ≥160 mg·dL^−1^ [18]. Hypertension was defined according to the self-reported physician diagnosis of hypertension, the use of anti-hypertensive medication, or systolic/diastolic blood pressure ≥130/80mmHg [19]. Smoking status was classified into three categories: never smoker, former smoker, and current smoker. Heavy alcohol drinking was defined as an average intake of >7 drinks/week for women and >14 drinks/week for men [20].

### Patient and public involvement

The patient and public were not involved in the study design, conduct of the study or plans to disseminate the results to study participants.

### Statistical analyses

Baseline characteristics are presented by those with and without a diverticulitis diagnosis, tertiles of CRF and BMI classification. Baseline differences in continuous variables were assessed using general linear model F-tests, while differences in categorical variables were assessed using Pearson’s chi-squared test (χ^2^).

Multivariate logistic regression was used to calculate the odds ratios (ORs) and 95% confidence intervals (CIs) of diverticulitis prevalence across the tertiles of CRF and BMI classification, with the lowest tertile of CRF and normal BMI serving as the reference groups, respectively. Analyses were adjusted for sex and age (years) in Model 1. Model 2 was adjusted for Model 1 plus smoking status (never, former, current), heavy alcohol drinking (yes or no), physical activity (steps/day), meals/week (quintiles), vegetable cups/day (quintiles), fruit cups/day (quintiles), non-steroidal anti-inflammatory drug use (yes or no), hypercholesterolemia (yes or no), and hypertension (yes or no). Model 3 was additionally adjusted for BMI (kg/m^2^) or CRF (minutes) for each other as a continuous variable. We also examined odds ratios of diverticulitis per minute increment of walking time and per kg/m^2^ increment of BMI using a continuous variable of CRF and BMI, respectively.

A joint analysis of CRF and BMI on diverticulitis was conducted. CRF was dichotomized into either “unfit” (e.g., lower one third) or “fit” (e.g., upper two thirds) to preserve statistical power and simplify the complicated joint association following earlier studies [21–23]. BMI was dichotomized into “normal weight” or “overweight/obese" following the WHO definition [14]. From this, following four categories were created: “unfit and overweight/obese,” “fit and overweight/obese,” “unfit and normal weight,” and “fit and normal weight” to calculate ORs (95% Cis) of diverticulitis across these combined categories of CRF and BMI. "Unfit and overweight/obese" was used as the reference group.

A sensitivity analysis was conducted to assess the robustness of the original statistical model by altering exposure categories. CRF was re-categorized from tertiles into quartiles to explore exposure–outcome relationships.

All analyses were conducted using SAS software version 9.4 (SAS Institute, Inc., Cary, NC). *P* values are two-sided, with *P* ≤ 0.05 regarded as significant.

## Results

There were 35 (7.4%) reported cases of diverticulitis among the 476 participants. Table 1 shows the baseline characteristics of the participants by tertiles of CRF and BMI. Participants in the upper CRF were more likely to be younger and more active than those with lower CRF.

**Table 1:** Baseline characteristics by cardiorespiratory fitness and body mass index categories.

		Tertiles of Cardiorespiratory Fitness				Body Mass Index			
Characteristic	All	Lower	Middle	Upper	*P* value	Normal Weight (<25.0 kg/m^2^)	Overweight (25.0–29.9 kg/m^2^)	Obese (≥30.0 kg/m^2^)	*P* value
N	476	156	163	157		169	187	120	
Female, n (%)	293 (61)	95 (60)	103 (63)	95 (60)	0.867	123 (72)	109 (58)	61 (50)	**< .0001**
Age (years)	71.2 (5.4)	74.1 (5.9)	70.7 (4.9)	68.8 (3.8)	**< .0001**	71.8 (5.7)	71.2 (5.3)	70.3 (5.0)	0.084
Body mass index (kg/m^2^)^a^	27.2 (4.7)	29.1 (5.3)	27.4 (4.2)	25.2 (3.7)	**< .0001**	22.7 (1.8)	27.3 (1.4)	33.5 (3.3)	**< .0001**
Physical activity (steps/day)	5684 (2998)	4503 (2475)	5858 (2859)	6674 (3220)	**< .0001**	5930 (2928)	5828 (3126)	5110 (2836)	0.050
Cardiorespiratory fitness (min)^b^	4.5 (0.8)	5.3 (0.8)	4.4 (0.2)	3.8 (0.3)	**< .0001**	4.3 (0.6)	4.4 (0.6)	4.9 (1.0)	**< .0001**
Smoking status, n (%)					0.488				0.387
Never smoked	325 (68)	102 (65)	113 (69)	110 (70)		114 (67)	133 (71)	78 (65)	
Former Smoker	146 (30)	51 (32)	48 (29)	47 (29)		52 (30)	54 (28)	40 (33)	
Current smoker	5 (1)	3 (1)	2 (1)	0 (0)		3 (1)	0 (0)	2 (1)	
Heavy alcohol drinking, n (%)^c^	39 (8)	15 (9)	11 (6)	13 (8)	0.646	15 (8)	14 (7)	10 (8)	0.891
Meal intake (meals/week)	19.6 (4.)	19.5 (3.0)	19.5 (2.4)	19.9 (5.8)	0.495	20.0 (2.7)	19.1 (4.9)	20.0 (3.9)	**0.045**
Vegetable intake (cups/day)	2.1 (3.5)	2.1 (3.1)	2.2 (4.9)	2.1 (2.0)	0.958	2.1 (1.7)	2.5 (5.3)	1.7 (1.1)	0.192
Fruit intake (cups/day)	2.2 (4.7)	2.1 (3.4)	2.4 (7.0)	2.1 (2.1)	0.767	2.0 (1.1)	2.7 (7.3)	1.7 (1.2)	0.127
Use of NSAIDs, n (%)^d^	104 (21)	44 (28)	34 (20)	26 (16)	**0.042**	21 (12)	49 (26)	34 (28)	**0.001**
Hypercholesterolemia, n (%)^e^	240 (50)	88 (56)	83 (50)	69 (43)	0.087	67 (39)	102 (54)	71 (59)	**0.002**
Hypertension, n (%)^f^	261 (55)	101 (65)	94 (58)	66 (42)	**< .0001**	70 (41)	109 (58)	82 (68)	**< .0001**

Data are presented as mean (SD) unless indicated otherwise.

^a^ Weight in kilograms divided by height in meters squared.

^b^ Cardiorespiratory fitness is time to complete a 400-meter walk test.

^c^ Heavy drinking is >7 drinks/week for women and >14 drinks/week for men.

^d^ Taking non-steroidal anti-inflammatory drugs (NSAIDs), such as aspirin or ibuprofen.

^e^ Defined as systolic/diastolic blood pressure ≥ 130/80mmHg, self-reported diagnosed hypertension, and/or taking blood pressure medication.

^f^ Defined as low-density lipoprotein cholesterol ≥160 mg·dL^−1^, self-reported diagnosed high cholesterol, and/or taking cholesterol medication.

Table 2 shows the ORs (95% CIs) of the prevalence of diverticulitis across tertiles of CRF and the three BMI categories. Compared to lower CRF, the OR (95% CI) for having diverticulitis in the upper CRF tertile was 0.30 (0.11–0.80) after controlling for age and sex (Model 1) and remained significant after further adjusting for lifestyle factors and medical conditions (Model 2). These associations were, however, no longer significant after further adjustment for BMI (Model 3). Compared to normal weight, the ORs (95% CIs) of diverticulitis was 2.85 (1.09–7.46) in the overweight group and 3.24 (1.16–9.05) in the obese group, respectively (Model 1). The association remained significant in the overweight group after further adjusting for lifestyle factors and medical conditions (Model 2), as well as CRF (Model 3). In the obese group, however, the association was no longer significant after adjustment for covariates or further adjustment for CRF. In the sensitivity analysis, ORs for diverticulitis were similar to using tertiles of CRF. Compared to quartile 1 (least fit), the ORs (95% CIs) for CRF quartiles in the fully adjusted model were 0.42 (0.15–1.28), 0.80 (0.29–2.21), and 0.39 (0.11–1.45), respectively, in quartiles 2, 3, and 4 (most fit).

**Table 2:** Odds ratios (ORs) and 95% confidence intervals (95% CIs) for diverticulitis according to cardiorespiratory fitness and body mass index.

			Model 1	Model 2	Model 3
	n	Cases	OR (95% CI)	OR (95% CI)	OR (95% CI)
**Tertiles of Cardiorespiratory Fitness**					
Lower CRF	156	17	1.00 (reference)	1.00 (reference)	1.00 (reference)
Middle CRF	163	11	0.50 (0.22–1.15)	0.52 (0.22–1.22)	0.55 (0.23–1.33)
Upper CRF	157	7	**0.30 (0.11–0.80)**	**0.33 (0.12–0.94)**	0.37 (0.12–1.10)
*P for linear trend*			**0.013**	**0.033**	0.067
Per minute in walking time			1.18 (0.75–1.86)	1.06 (0.64–1.78)	0.89 (0.49–1.63)
**Body Mass Index**					
Normal weight (<25.0 kg/m^2^)	169	6	1.00 (reference)	1.00 (reference)	1.00 (reference)
Overweight (25.0–29.9 kg/m^2^)	187	17	**2.85 (1.09–7.46)**	**2.83 (1.04–7.66)**	**2.86 (1.05–7.79)**
Obese (≥30.0 kg/m^2^)	120	12	**3.24 (1.16–9.05)**	2.83 (0.96–8.38)	2.98 (0.95–9.35)
*P for linear trend*			**0.024**	0.068	0.063
Per kg/m^2^ in body mass index			1.06 (0.99–1.14)	1.05 (0.98–1.13)	1.06 (0.97–1.15)

Model 1 was adjusted for sex and age (years).

Model 2 was adjusted for Model 1 plus smoking status (never, former, current), heavy alcohol drinking (yes or no), physical activity (steps/day), meals/week (quintiles), vegetable cups/day (quintiles), fruit cups/day (quintiles), non-steroidal anti-inflammatory drugs use (yes or no), hypercholesterolemia (yes or no), hypertension (yes or no).

Model 3 was adjusted for Model 2 plus body mass index (kg/m^2^) for CRF or CRF (min) for body mass index.

**Fig 1:**
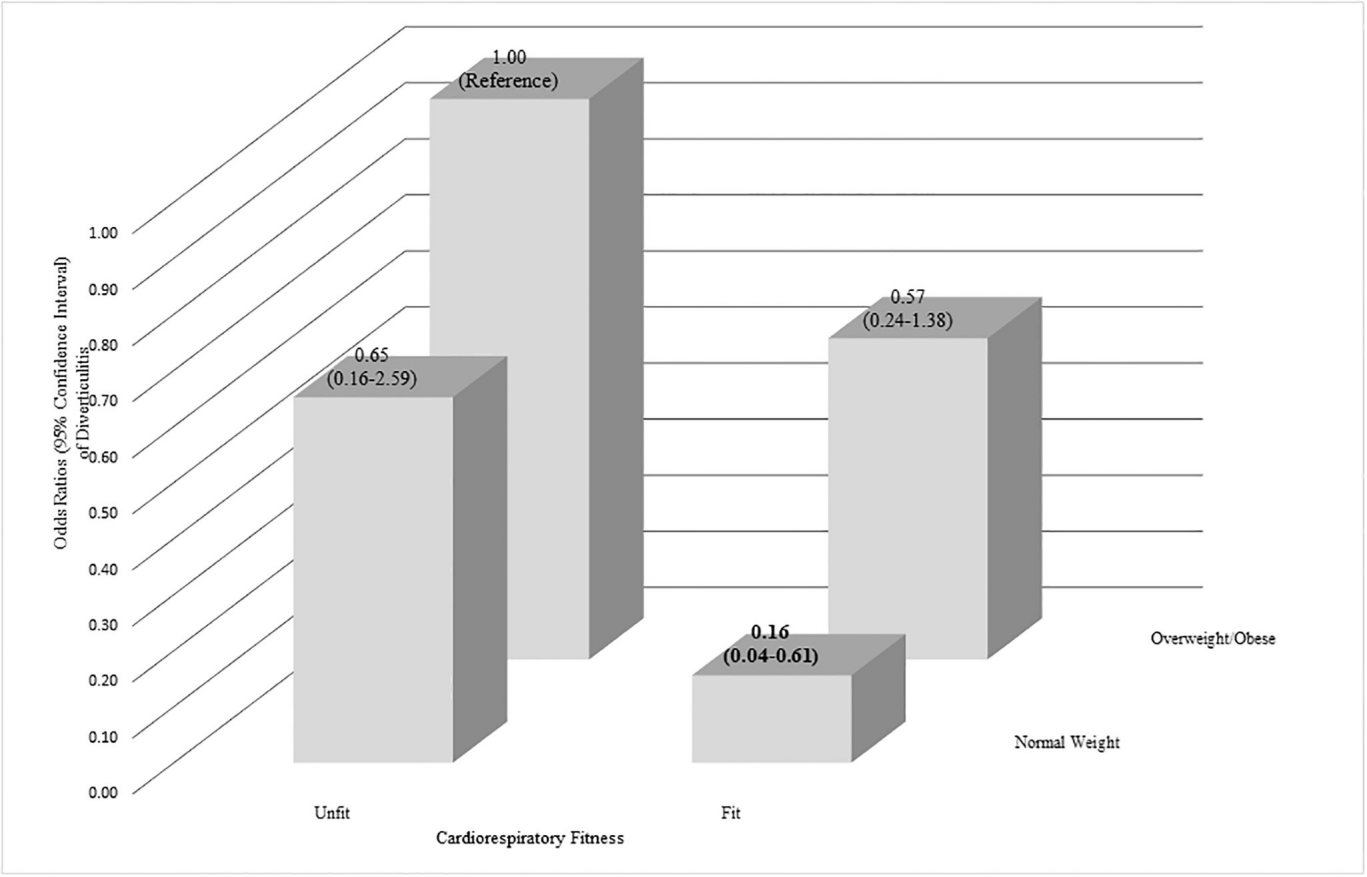
Joint associations of cardiorespiratory fitness and body mass index with diverticulitis.

Fig 1 shows the results from the joint analysis of CRF and BMI on diverticulitis. Compared to the “unfit and overweight/obese” group, the “fit and overweight/obese,” “unfit and normal weight,” and “fit and normal weight” groups had ORs (95% CIs) of 0.57 (0.24–1.38), 0.65 (0.16–2.59), and 0.16 (0.04–0.61), respectively.

Participants were divided into four groups based on combined categories of cardiorespiratory fitness (unfit or fit) and body mass index (normal-weight, overweight/obese), respectively. "Unfit" was the lower third of cardiorespiratory fitness and "fit" was the upper two thirds of cardiorespiratory fitness. Normal weight was body mass index < 25.0 kg/m^2^, overweight/obese was ≥ 25.0 kg/m^2^. The model was adjusted for sex, age (years), smoking status (never, former, current), heavy alcohol drinking (yes or no), physical activity (steps/day), meals/week (quintiles), vegetable cups/day (quintiles), fruit cups/day (quintiles), non-steroidal anti-inflammatory drugs use (yes or no), hypercholesterolemia (yes or no), and hypertension (yes or no). The number of participants (cases of diverticulitis) in the “overweight/obese and unfit,” “overweight/obese and fit,” “normal weight and unfit,”, and “normal weight and fit” groups were 117 (14), 190 (15), 39 (3), and 130 (3), respectively.

## Discussion

The primary findings of this investigation were: 1) high CRF is associated with reduced odds of diverticulitis, independent of potential confounders; however, the association was no longer significant after further adjustment for BMI (Table 2); 2) overweight is associated with an increased prevalence of diverticulitis even after adjusting for potential confounders including CRF (Table 2); and 3) high CRF with lower BMI is significantly associated with lower odds of diverticulitis, suggesting potential additive benefits in the combination of high CRF and lower BMI in relation to the prevalence of diverticulitis in older adults (Fig 1).

Limited research exists on the relationship between CRF and diverticulitis with only one previous study assessing the relationship between CRF and diverticulitis. In the prospective National Runners’ Study, which included 10,776 men and 1,939 women aged 50 years and older [8], Williams found that greater CRF, assessed by self-reported 10 km race speed, was inversely related to the risk for self-reported incident diverticulitis. Clinically, the risk reduction was substantial, 6.2% per km/day of running, resulting in a 48% lower incidence in those who ran ≥8 km/day relative to those who averaged between 0 and 2 km/day (*P* = 0.05). However, adjustment for baseline BMI diminished the significance of the risk reduction (*P* = 0.08). This result is similar to our cross-sectional finding that higher CRF was no longer significantly associated with lower prevalence of diverticulitis after adjusting for BMI (Table 2). However, the previous study was conducted in a younger population (≥50 years) and CRF was defined differently using self-reported running race speed, which should be considered in the interpretation of the data.

The mechanisms by which CRF affects diverticulitis pathogenesis are not known. CRF may enhance colonic motility, which is generally accepted to be related to gastrointestinal disorders [24]. The relationship between PA and diverticulitis, however, has been more thoroughly investigated. Higher PA is thought to lower the risk of diverticulitis by reducing body weight and BMI, maintaining gastrointestinal motility, decreasing intra-colonic pressure, reducing the food transit time, and through neuroendocrine changes [25]. Both PA and CRF are associated with lower obesity-related metabolic risk factors such as elevated triglycerides, hypertension, hyperglycemia, insulin resistance [21, 26], and abdominal adiposity, a known contributor to the development of diverticulitis. Given that higher CRF is largely attributable to higher PA levels, the mechanisms by which CRF influences diverticulitis may be similar to that of PA. Other potential mechanism includes increased inflammation due to obesity and/or reduced PA in the elderly given both increase diverticulitis [6, 25, 27, 28]. One previous study [9], a meta-analysis of PA in relation to the risk of diverticulitis, reported that there was a 26% reduction in the risk of diverticulitis for the highest PA compared to the lowest level of PA. Through these previous results, it is plausible that higher CRF may significantly reduce the prevalence and potentially the risk of diverticulitis in older adults. However, prospective studies are required to confirm this.

BMI is a known risk factor for diverticulitis [6, 9, 10]. Our results suggesting an increased prevalence of diverticulitis with increasing BMI are therefore not surprising and align with a recent meta-analysis of prospective studies reporting a 31% increase in risk of diverticulitis for a 5-unit increase in BMI [9]. These findings suggest that reducing BMI might provide a protective effect against diverticulitis. We expanded upon the previous literature by further adjusting our analysis of BMI and diverticulitis by CRF. After adjustment for CRF, the odds of diverticulitis in the overweight group remained significant. Our joint analysis also showed a possible additive association of higher CRF and lower BMI on a reduced prevalence of diverticulitis (Fig 1) with the OR (95% CI) of 0.16 (0.04–0.61), which has not been explored in prior studies. This novel finding suggests that higher CRF combined with normal body weight significantly reduces the prevalence of diverticulitis in older adults.

There are several limitations to this study. Diverticulitis was not ascertained or confirmed by medical tests (e.g., gastrointestinal endoscopy, stool test, or computed tomography scan); rather, it was based on physician diagnosis using a standardized medical history questionnaire. Thus, misclassification is possible although the objective medical tests to diagnose diverticulitis are expensive and would be challenging in large cohort studies. Detailed information regarding diet (e.g., red meat intake, total energy intake, fiber, and water), which is another important factor related to diverticulitis, was unavailable. However, we adjusted for general dietary information such as meals/week, as well as fruit and vegetable intake which are likely markers of fiber intake (although other sources also may be important), to minimize the potential confounding effect of diet. Although ORs were lower in middle and upper CRF groups (ranging from 0.55 to 0.30) compared to the lower CRF group, and higher in overweight and obese BMI groups (ranging from 2.83 to 3.24) compared to the normal weight group, many ORs were not statistically significant. This result is possibly due to the small number of diverticulitis cases (n = 35) in this older adult cohort. Therefore, future studies with a larger sample size and more diverticulitis cases are clearly needed to confirm our findings. Lastly, given the cross-sectional analysis, we cannot determine whether the observed associations are causal, however, the results are consistent with data from large cohort studies. The results from this study may also vary in other older adult populations given our cohort is mostly white, highly educated, and living independently.

Despite these limitations, to our knowledge, this is the first study on the independent and joint associations of CRF and BMI with diverticulitis in older adults considering the various possible confounders in our data analyses, including demographic factors (sex and age), behavioral factors (smoking, alcohol intake, PA, and diet), medications and medical conditions (NSAIDs, hypercholesterolemia, and hypertension), which are well-established risk factors of diverticulitis. Our findings could provide important preliminary data to develop future prospective studies and guidelines in older adults who are at high risk of developing diverticulitis.

In conclusion, CRF and BMI appear to be associated with the prevalence of diverticulitis in older adults. Specifically, higher CRF combined with lower BMI is more strongly associated with a lower prevalence of diverticulitis.
